# Relationships Between Cell Structure Alterations and Berry Abscission in Table Grapes

**DOI:** 10.3389/fnut.2020.00069

**Published:** 2020-06-12

**Authors:** Mingjuan Li, Zhanwen Huang, Xiangrong You, Yayuan Zhang, Ping Wei, Kui Zhou, Ying Wang

**Affiliations:** ^1^Agro-Food Science and Technology Research Institute, Guangxi Academy of Agricultural Sciences, Nanning, China; ^2^Guangxi Key Laboratory of Fruits and Vegetables Storage-Processing Technology, Nanning, China; ^3^Flower Research Institute, Guangxi Academy of Agricultural Sciences, Nanning, China

**Keywords:** table grape, microstructure, ultrastructure, berry abscission, during storage

## Abstract

The aim of this study was to explore the relationships between structure alterations and postharvest berry abscission in “Muscat Kyoho” “Kyoho” and “Nanyu” table grapes stored for 0, 3, or 6 days at room temperature. Microstructure analysis showed that a large number of the stalk-berry junction cells of “Muscat Kyoho” and “Kyoho” were lignified and suberized at 0 day, whereas these events seldom occurred in “Nanyu.”Furthermore, the berry brush cells of the three varieties, especially those of “Nanyu,” were small and dense. At 3 days, the numbers of lignified and suberized cells of “Muscat Kyoho” and “Kyoho” were reduced, and the cells had degraded, ruptured, and disappeared by 6 days. The berry brush cells of “Muscat Kyoho” and “Kyoho” were larger and more loosely arranged than were those of “Nanyu.” Ultrastructure analysis showed that the cells increased in size from small to large and became loosely arranged; the smallest changes were observed in “Nanyu.” The cells of “Muscat Kyoho” and “Kyoho” were hydrolyzed, liquated, and covered by granular substances at 6 days, and these features were especially prominent in “Muscat Kyoho.” The detachment force of grapes declined steadily (*p* < 0.05) and was accompanied by an increase in berry abscission. “Nanyu” maintained the highest detachment force and the lowest berry abscission during storage (*p* < 0.05), followed by “Kyoho” and “Muscat Kyoho.” Structural alterations were directly related to berry abscission and correlated inversely with detachment force, with the greatest alterations occurring in “Muscat Kyoho,” followed by “Kyoho” and then “Nanyu.”

## Introduction

The table grape (*Vitis vinifera* L.) is popular with consumers because of its color, shape, taste, and high nutrient content. However, its rapid deterioration in the absence of appropriate treatments and its short postharvest life limit its consumption (1, 2). The grape is thin-skinned, soft, and succulent and lacks a hard shell for protection, and its metabolism is very active during postharvest storage. These traits can lead to many undesirable phenomena, such as rachis browning, susceptibility to disease, rapid aging, fungal rot, berry abscission, moisture loss, softening, and alterations in flavor, which severely restrict the storage and preservation of table grapes (3–5). Berry abscission is the process in which plant tissues or organs separate from the body of a plant; it is a common phenomenon in plant life cycles (6, 7). Berry abscission (berry drop) of table grapes can be divided into three categories: (1) berry shatter, in which the berries are sloughed from the cap stem due to the fragile tissue structure of the stalk (8); (2) wet drop, in which the berries become detached from the stems, and the berry brushes remain attached to the pedicel (9); and (3) dry drop, in which an abscission layer zone forms at the berry–pedicel or stalk–pedicel junction, and the berry brushes remain attached to the berry (10). Berry abscission during storage and transport represents a frequent, serious problem in the marketing of table grapes.

In recent years, numerous studies have shown that the berry abscission of grapes is associated with a variety of factors, such as abscission-related factors during berry development (11), fruit detachment force (12), ultrastructural changes in abscission zones (13), hormone type and content (14), enzyme activity in the abscission zone (2, 15, 16), and plant growth regulator application (17, 18). Berry abscission in “Kyoho” grapes usually occurs as dry drop; in this process, decreasing levels of ethylene and auxin induce the formation of an abscission zone at the stalk–berry junction (19–21). Deng et al. (8) reported the postharvest berry abscission of “Kyoho” table grapes during cold storage and in high-oxygen atmospheres while investigating the best storage method to extend shelf life. However, berry abscission in “Muscat Kyoho” and “Nanyu” grapes has been less well studied.

Although the berry abscission of table grapes has been studied extensively, studies of the mechanism of berry abscission from the perspective of microstructure, ultrastructure, berry abscission, and detachment force of table grapes during storage at room temperature are scarce. In previous studies, table grapes were normally stored at low temperatures in combination with a gaseous treatment. Nevertheless, the table grapes picked from vineyards by consumers are usually stored at room temperature and are ready to eat, representing a common method for picking and eating grapes in the region. Regardless, berry abscission is a frequent phenomenon, and the storage period is usually no more than 7 days at room temperature. Therefore, in our study, we performed experiments at room temperature and made observations every 3 days. Our purpose was to simulate the shelf life test at room temperature. In this study, three table grape cultivars, namely, “Muscat Kyoho,” “Kyoho,” and “Nanyu,” were stored at room temperature (25 ± 1°C) for 0, 3, or 6 days after harvest. Scanning electron microscopy (SEM) was then used to examine the microstructure of the stalk–berry junction and berry brush, and the ultrastructures of the pedicel, stalk–berry junction, and berry brush were scanned and compared among the time points and cultivars. The changes in the microstructure and ultrastructure of the berries among the different storage periods were studied to explore the causes of berry abscission from a cytological perspective.

## Materials and Methods

### Materials and Treatment

Three table grape varieties “Muscat Kyoho,” “Kyoho,” and “Nanyu,” which mature in July (soluble solid content higher than 16%), were used as the test materials. All of the table grapes were collected from the vineyard of Guangxi Academy of Agricultural Sciences located in Nanning, Guangxi, China. Clusters were selected based on uniform maturity, size, hardness, firmness, and the absence of blemishes or disease. After picking, the clusters were immediately transported to the Guangxi Key Laboratory of Fruits and Vegetables Storage-Processing Technology within 2 h. All of the table grape bunches were stored at 25 ± 1°C in approximately 85% relative humidity for 0, 3, or 6 days until analysis.

### Preparation of Grape Fruit Tissue for Microscopic Structural Observation

The specimens of table grapes were prepared for microscopic structural observation by embedding them in paraffin following the procedure of Deng et al. (8). The process was as follows: 3~5 mm^3^ of tissue from the fruit stalk, including the berry brush, was excised quickly with a fresh razor blade. The tissue sample was fixed immediately in precooled FAA solution (70% ethanol:38% formaldehyde:acetic acid; 90:5:5, v:v:v) for 24 h at 4°C. The samples were then dehydrated with a graded alcohol series (30, 50, 70, 80, 90, 95, and 100%), cleared with transparent reagent, embedded in paraffin, sliced into 8- to 12-μm sections on a rotator microtome, stained with safranin and fast green, and routinely examined using a fluorescence microscope (Leica DM4000B, Germany). More than eight sections per sampling time were prepared for observation.

### Preparation of Grape Fruit Tissue for Ultrastructural Observation

**Figure 1:**
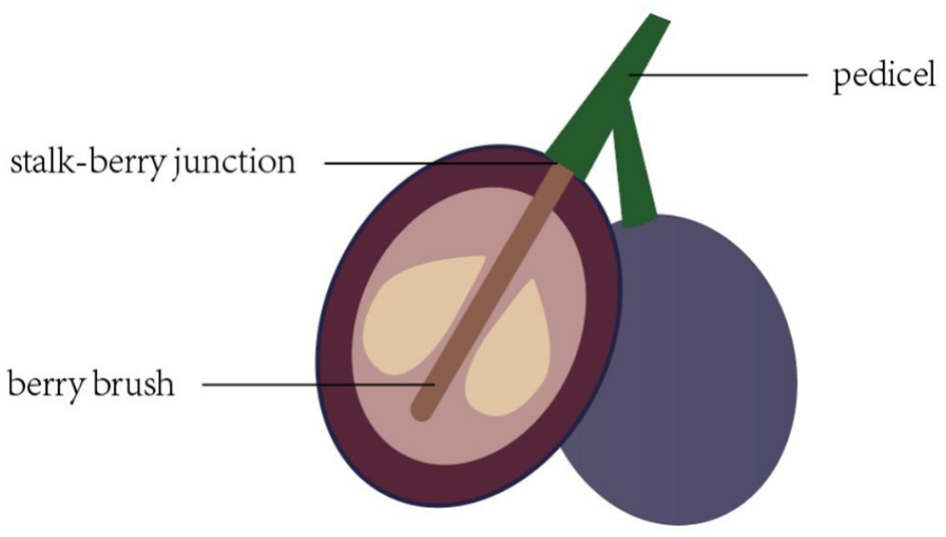
Stalk–berry junction, berry brush, and pedicel of the grape berry and sampling site used for cell structure determination.

The ultrastructure of the table grapes was examined using SEM. The method described by Casado and Heredia (22) was used to prepare the tissue; the process was as follows: 3~5 mm^3^ of tissue from the fruit pedicel, stalk–berry junction, and berry brush was excised quickly with a sharp razor blader (Figure 1). The tissue sample was fixed in precooled glutaraldehyde fixative (2.5% v v^−1^ in 0.1 mol L^−1^ phosphate buffer, pH = 7.2) for 24 h at 4°C and postfixed with OsO_4_ (2% v v^−1^ in the same buffer) for 4 h at 4°C. The specimens were then thoroughly rinsed in fresh phosphate buffer solution and dehydrated with a graded ethanol series (30, 50, 70, 80, 90, 95, and 100%) for increasing amounts of time from 15 to 120 min. Then, the samples were sliced, dried, placed on a metallic holder using a double-faced adhesive, and sprayed with gold dust. Finally, observation and photography were carried out with a scanning electron microscope (TESCAN Vega 3 LMU, Europe).

### Berry Abscission of Grape

Three clusters of each variety were used to examine berry abscission during storage. The berry abscission of table grapes was recorded using a scale with an accuracy of 0.01 g (JA2002, China) and expressed as a percentage of initial weight.

### Detachment Force of Grape

The detachment force of grapes was determined using a CT_3_ Texture Analyzer (BROOKFIELD, USA). An individual grape pedicel was passed through the hole of a homemade fixed plate, and the grape berry was fixed firmly at the pedicel with a clamp. The clamp was used to fasten the texture analyzer to the top of the grape pedicel. The texture analyzer was moved upward until the pedicel was detached from the berry. The maximum force was the detachment force during the tension test. This test was performed at a speed of 2 mm/s, the pretest and posttest speeds were 3 mm/s, and a trigger force of 5 g was used. Twenty grape berries with pedicels attached were measured each time for each variety.

### Statistical Analysis

The data were analyzed using SPSS 19.0 statistical data analytical software (SPSS Inc., Chicago, IL, USA). Results are presented as the mean ± standard deviation (SD). Mean differences were established by the Duncan's multiple comparison test (*p* < 0.05).

## Results

### Microstructure Alterations of Table Grapes During Storage

#### Cell Microstructure Alterations of the Stalk–Berry Junction

**Figure 2:**
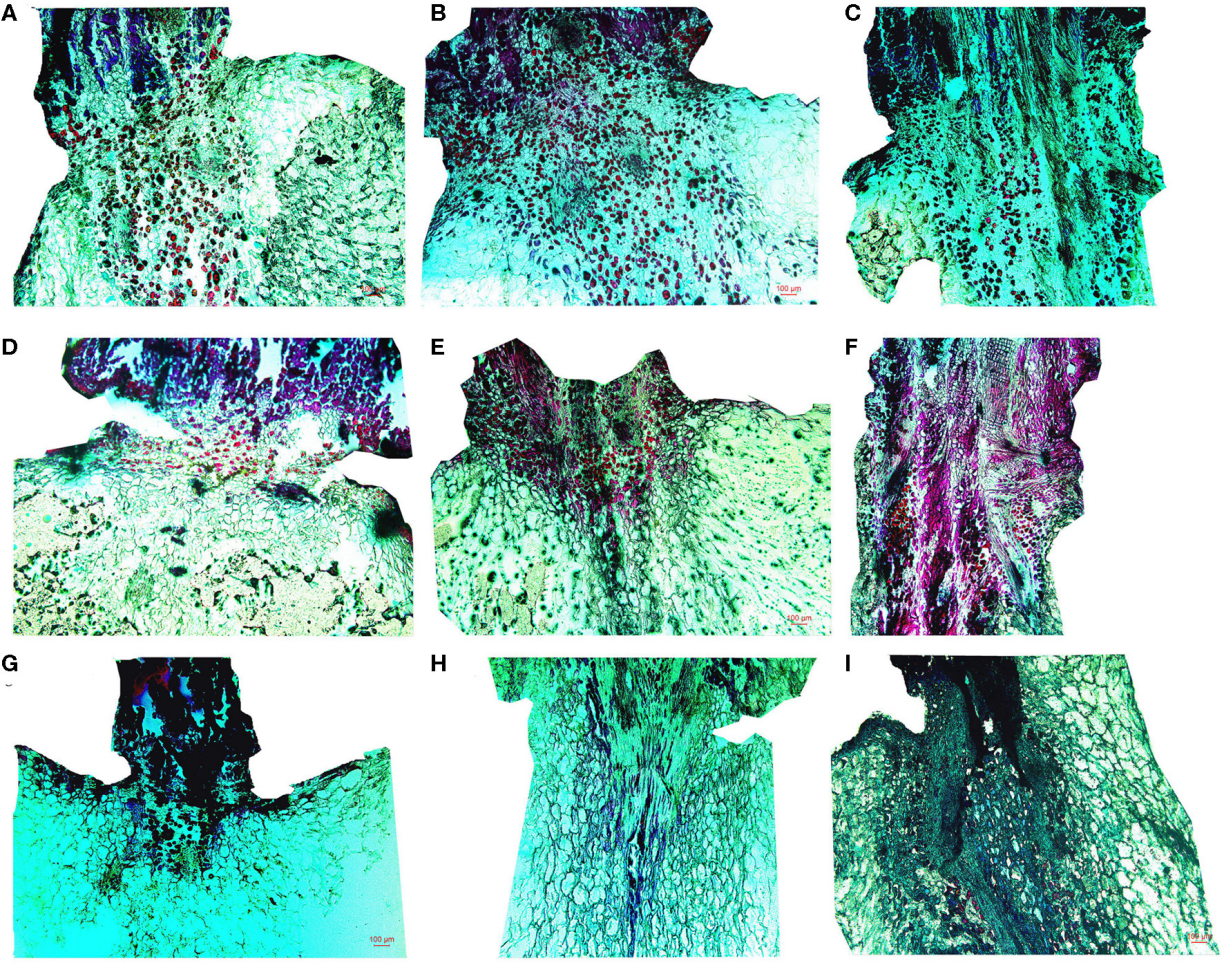
The microstructure of the stalk–berry junction cells of table grapes stored for 0, 3, or 6 days (×50). After storage for 0 day in “Muscat Kyoho” **(A)**, “Kyoho” **(B)**, and “Nanyu” **(C)**; after storage for 3 days in “Muscat Kyoho” **(D)**, “Kyoho” **(E)**, and “Nanyu” **(F)**; after storage for 6 days in “Muscat Kyoho” **(G)**, “Kyoho” **(H)**, and “Nanyu” **(I)**. All images are of transverse sections.

The cells at the stalk–berry junction of the three table grape cultivars all underwent a natural sequence of changes during storage; selected micrographs representing the trend of the microstructure changes are presented in Figure 2. With 0 day of storage, large numbers of the cells at this junction in “Muscat Kyoho” and “Kyoho” were stained red or black, indicating that these cells were lignified and suberized, and the cells destined to be involved in berry abscission were not yet discernible (Figures 2A,B). In contrast, cells stained red were less visible in “Nanyu” (Figure 2C). After storage for 3 days, the numbers of lignified and suberized cells in “Muscat Kyoho” and “Kyoho” were reduced (Figures 2D,E), but the number had increased significantly in “Nanyu” (Figure 2F). After storage for 6 days, the lignified and suberized cells of the three varieties had degraded and disappeared completely (Figures 2G–I). The cells of “Muscat Kyoho” were disintegrated, resulting in partial intercellular cavities, and the berries ultimately separated from the stalk–berry junction (Figure 2G). Some cells of “Kyoho” began to dissolve and separate, forming intercellular cavities (Figure 2H). The cells of “Nanyu” were tightly arranged (Figure 2I).

#### Cell Microstructure Alterations of the Berry Brush

**Figure 3:**
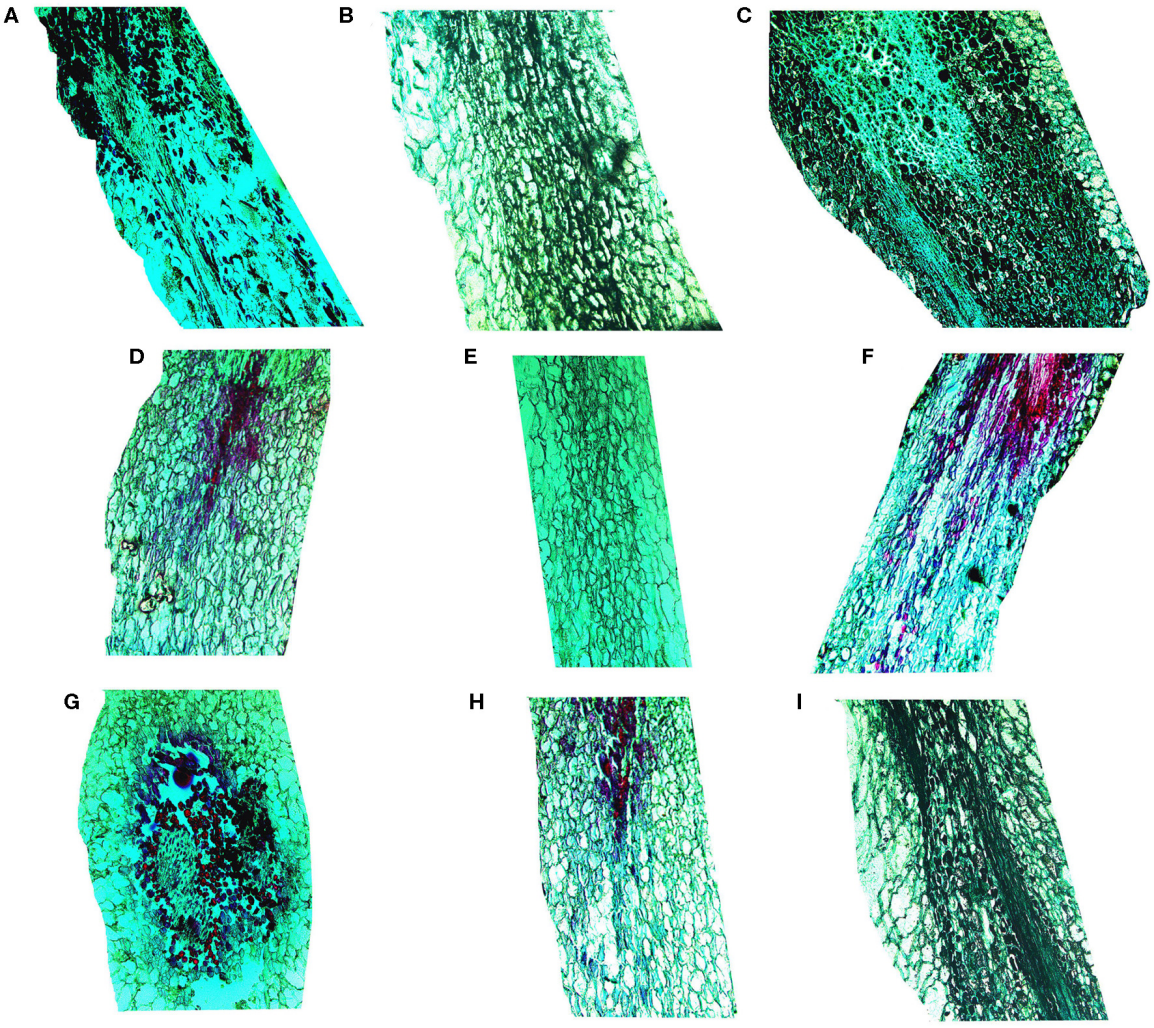
The microstructure of berry brush cells of table grapes stored for 0, 3, or 6 days (×50). After storage for 0 day in “Muscat Kyoho” **(A)**, “Kyoho” **(B)**, and “Nanyu” **(C)**; after storage for 3 days in “Muscat Kyoho” **(D)**, “Kyoho” **(E)**, and “Nanyu” **(F)**; after storage for 6 days in “Muscat Kyoho” **(G)**, “Kyoho” **(H)**, and “Nanyu” **(I)**. All images are of transverse sections.

Micrographs of the berry brush of table grapes are shown in Figure 3. Compared with those of “Muscat Kyoho,” the berry brush cells of “Kyoho” and “Nanyu” were smaller and arranged more compactly at 0 day of storage, especially those of “Nanyu” (Figures 3A–C). At 3 days of storage, the berry brush cells of the three varieties were more loosely arranged, the intercellular spaces were larger, and cytoplasm–cell wall separation was observed (Figures 3D–F). The berry brush cells of “Muscat Kyoho” and “Nanyu” were stained red or black, indicating that they were lignified and suberized (Figures 3D,F). At 6 days of storage, severe microstructural damage was apparent in the berry brush of “Muscat Kyoho,” and the cells had dissolved and separated, leading to intercellular cavities (Figure 3G). The cytoplasm–cell wall separation was extensive in the berry brush of “Kyoho,” in which the cells were loosely arranged, partially lignified and suberized (Figure 3H). The berry brush cells of “Nanyu” were complete relative to those of the other two varieties, and the lignified and suberized cells had degraded and had disappeared (Figure 3I).

### Ultrastructure Alterations of Table Grapes During Storage

#### Cell Ultrastructure Alterations of the Fruit Pedicel

**Figure 4:**
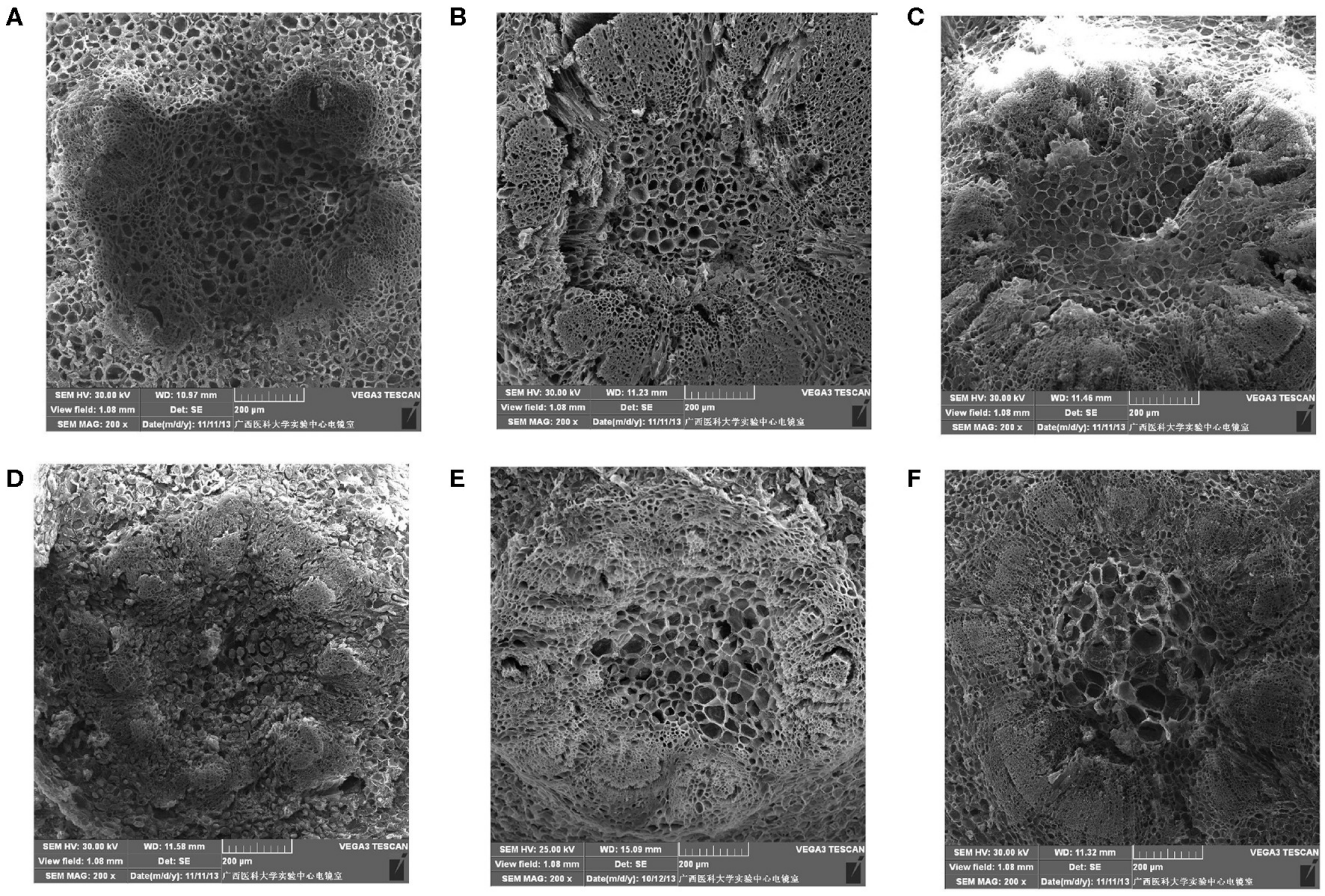
The ultrastructure of fruit pedicel cells of table grapes in storage for 0 or 6 days (×200). After storage for 0 day in “Muscat Kyoho” **(A)**, “Kyoho” **(B)**, and “Nanyu” **(C)**; after storage for 6 days in “Muscat Kyoho,” **(D)**, “Kyoho,” **(E)**, and “Nanyu” **(F)**. All images are of vertical sections.

The fruit pedicel ultrastructure of the table grapes is shown in Figure 4. At 3 days of storage, the vascular bundle cells of the fruit pedicel of the three kinds of table grapes were small and compactly arranged, and stone cells were arranged tightly around the vascular bundle (Figures 4A–C). After storage for 6 days, changes in the dispersion of the cells of the fruit pedicel vascular bundle compared to that observed at 0 day were detected, and a few granule cells were observed in “Muscat Kyoho” tissue, causing the size and outline of cells to be unclear (Figure 4D). The size and distribution of the vascular bundle cells of “Kyoho” and “Nanyu” showed variation in the extent of uniformity, with larger and less compactly arranged cells than observed at 3 days, whereas the stone cells of “Nanyu” were smaller and more compactly arranged than those of “Kyoho” (Figures 4E,F).

#### Cell Ultrastructure Alterations of the Stalk–Berry Junction

**Figure 5:**
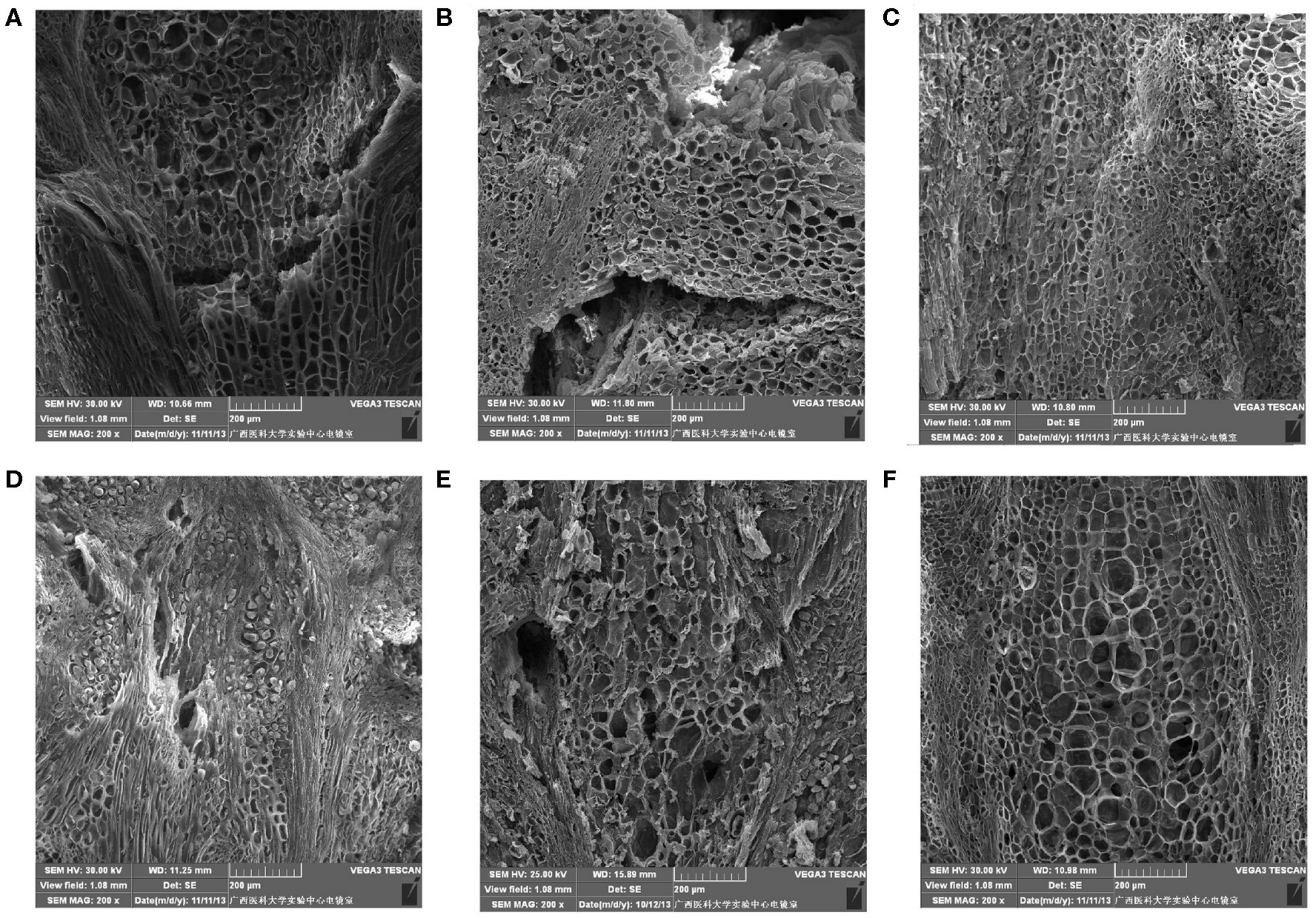
The ultrastructure of stalk–berry junction cells of table grapes after storage for 0 or 6 days (×200). After storage for 0 day in “Muscat Kyoho” **(A)**, “Kyoho” **(B)**, and “Nanyu” **(C)**; after storage for 6 days in “Muscat Kyoho” **(D)**, “Kyoho” **(E)**, and “Nanyu” **(F)**. All images are of transverse sections.

Figure 5 shows that after storage for 0 day, the cells at the stalk–berry junction of “Muscat Kyoho” were much larger than those of the other varieties and that those of “Kyoho” were larger than those of “Nanyu,” with the cells of “Nanyu” being small and tightly arranged (Figures 5A–C). After storage for 6 days, many granule cells were present at this junction in “Muscat Kyoho,” and the associated ultrastructural changes caused disarray and blurriness of the cell arrangement (Figure 5D). In “Kyoho,” a few granule cells and larger, less turgid cells than in the other varieties were observed (Figure 5E). In contrast, granule cells were not found in “Nanyu” (Figure 5F), and the ultrastructure lesions were less severe in this variety than in “Muscat Kyoho” and “Kyoho.”

#### Cell Ultrastructure Alterations of the Berry Brush

**Figure 6:**
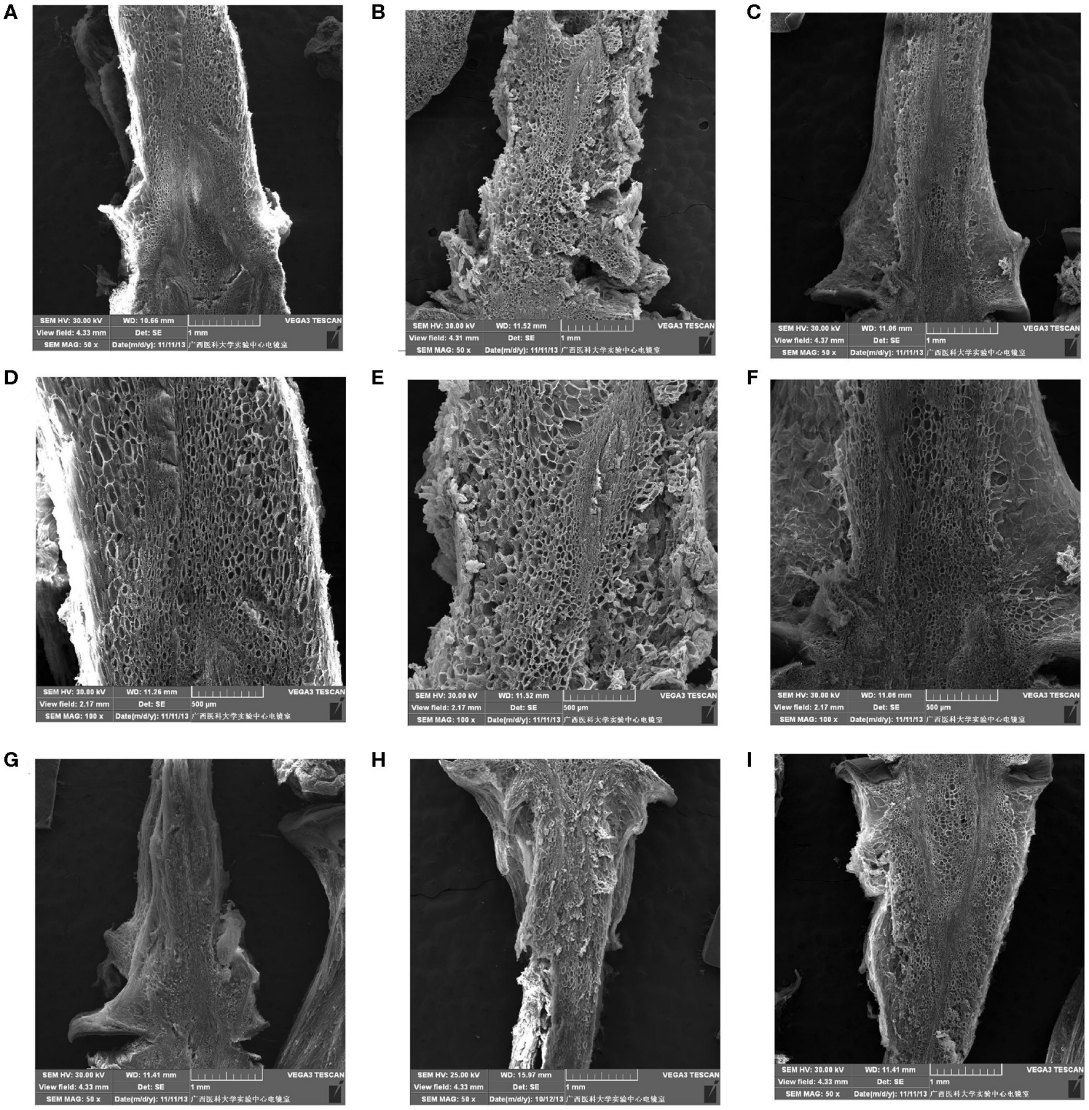
The ultrastructure of berry brush cells of table grapes stored for 0 or 6 days. After storage for 0 day in “Muscat Kyoho” (×50) **(A)**, “Kyoho” (×50) **(B)**, and “Nanyu” (×50) **(C)**; after storage for 0 day in “Muscat Kyoho” (×100) **(D)**, “Kyoho” (×100) **(E)**, and “Nanyu” (×100) **(F)**; after storage for 6 days in “Muscat Kyoho” (×50) **(G)**, “‘Kyoho” (×50) **(H)**, and “Nanyu” (×50) **(I)**. All images are of transverse sections.

As shown in Figure 6, after storage for 0 day, the berry brush cells of “Muscat Kyoho” and “Kyoho” were larger and less compactly arranged than those of “Nanyu” (Figures 6A,B,D,E). The berry brush cells of “Nanyu” were small, tightly appressed, highly organized, and closely associated with the sarcocarp cells (Figures 6C,F). At 6 days of storage, the berry brush cells of “Muscat Kyoho” were hydrolytic, liquated, and covered by granular objects, causing ambiguity in the size and outline of the cells (Figure 6G). Compared with “Muscat Kyoho” and “Kyoho,” “Nanyu” maintained smaller cells with a more organized arrangement (Figure 6I).

### Berry Abscission of Table Grapes During Storage

**Figure 7:**
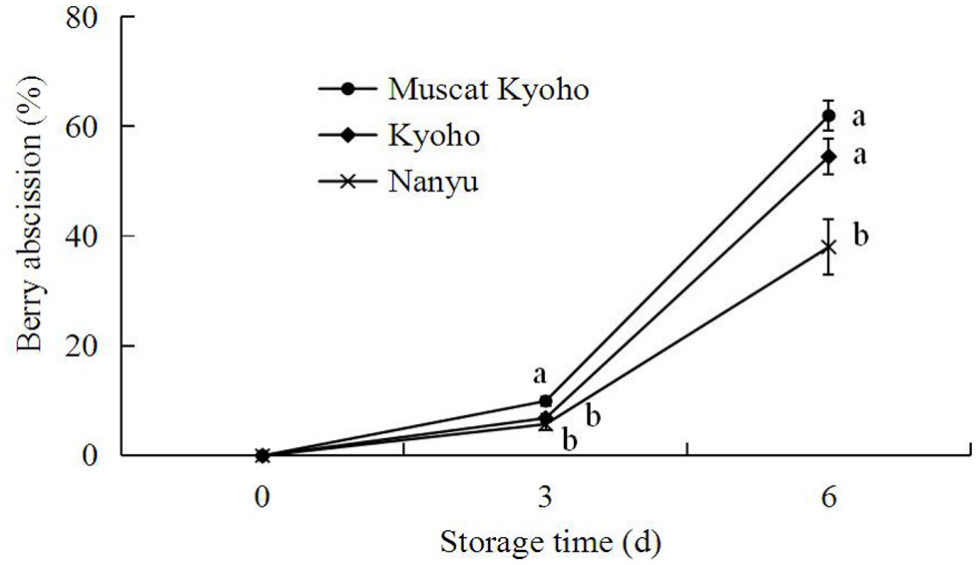
Changes in berry abscission of table grapes during storage. Data are means of three trials ± SD. Different lowercase letters in the same storage time indicate significant differences (*p* < 0.05).

As shown in Figure 7, berry abscission of table grapes increased gradually during the storage period. Additionally, “Muscat Kyoho” showed greater berry abscission than “Kyoho,” and “Kyoho” showed greater berry abscission than “Nanyu” throughout each experiment. At 3 days of storage, the berry abscission of “Muscat Kyoho” was significantly higher than that of “Kyoho” and “Nanyu” (*p* < 0.05), but there was no significant difference between “Kyoho” and “Nanyu” (*p* > 0.05). By day 6, the berry abscissions of “Muscat Kyoho” and “Kyoho” were not significantly different (*p* > 0.05), although they were significantly higher than that in “Nanyu” by 23.94 and 16.46% (*p* < 0.05), respectively.

### Detachment Force of Table Grapes During Storage

**Figure 8:**
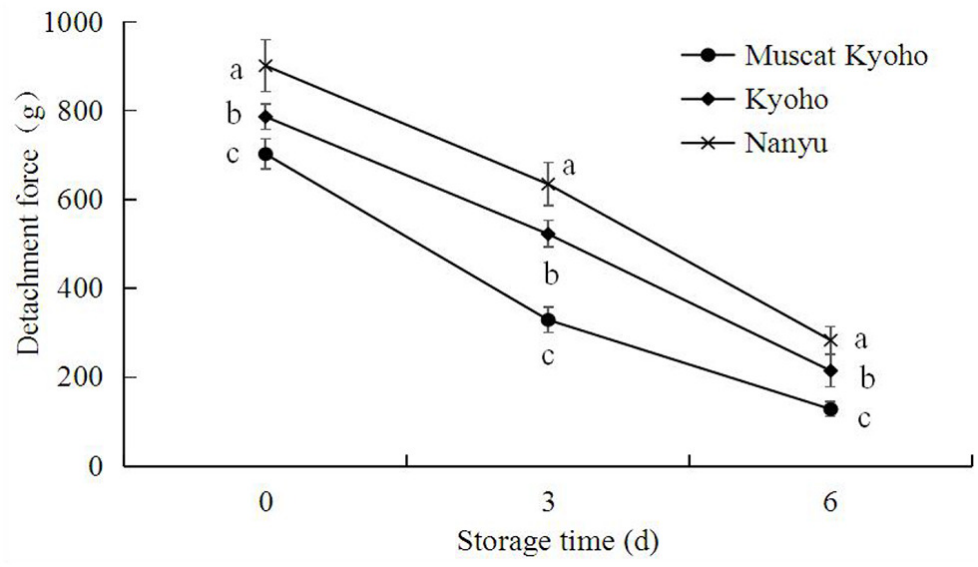
Changes in detachment force of table grapes during storage. Data are means of 20 trials ± SD. Different lowercase letters in the same storage time indicate significant differences (*p* < 0.05).

The detachment force of table grapes during storage is shown in Figure 8. With the extension of storage time, the detachment force of table grapes decreased steadily. The detachment forces of “Muscat Kyoho,” “Kyoho,” and “Nanyu” declined from 702.80, 786.60, and 901.40 g initially to 128.70, 215.15, and 283.80 g at the conclusion of the experiment, respectively. At the same storage time, the detachment force of “Nanyu” was higher than that of “Kyoho,” followed by that of “Muscat Kyoho,” and the differences were significant (*p* < 0.05).

## Discussion

The stalk–berry junction of table grapes plays an essential role in energy consumption after harvest. After harvest, physiological changes occur, and the metabolism remains active. The cell structure at the stalk–berry junction is closely related to berry abscission (23). The results presented here are similar to those reported by Deng et al. (8), who conducted a detailed histochemical study of grape berry abscission. The authors observed an increase in the size of stalk–berry junction cells in “Muscat Kyoho” and “Kyoho” with prolonged storage; in some cases, the cells dissolved and separated, forming partial intercellular cavities, which resulted in cell separation in the abscission zone (24).

Our results are in agreement with those of Deng et al. (8, 12), who reported a strong relationship between both the shape and size of the berry brush and berry abscission. Because the berry brush of “Kyoho” is short and small, the detachment force is weak during storage (25); therefore, the fruit resistance to abscission depends mainly on the tensile strength of the abscission zone. The marked decrease in fruit tensile strength during storage may be explained by the observed cell dissolution and separation in the berry brush; these results are consistent with those obtained for “Muscat Kyoho.”

The fruit pedicel is an important part of the berry–stalk connection; it is composed of vascular bundles and stone cells. During the preharvest period, the fruit pedicel plays a role in transporting water and nutrients into the berry. During the postharvest period, this function is ceased, respiration and transpiration increase, energy and water are consumed at high levels, and the berry becomes vulnerable to pathogenic microorganisms and pests. These phenomena are related to the abundant conducting tissue and loose arrangement of the fruit pedicel cells. Therefore, the fruit pedicel is closely associated with the storage characteristics of the fruit. Studies have shown that a larger fruit pedicel area is associated with stronger antivibration, mechanical damage, and support functions such that during the storage process, the berry is not easily abscised. Consistent with these observations, Zhou and Li (26) reported that the pedicel area of “Kyoho” was smaller than the pedicel areas of “Red Globe,” “Autumn Red,” and “Autumn Black” and that the berry of “Kyoho” was easier to abscise than were the berries of the other three varieties. In this study, we compared the ultrastructure of vascular bundle cells and stone cells among “Muscat Kyoho,” “Kyoho,” and “Nanyu,” and we demonstrated that a compact and uniformly distributed arrangement of cells increases the resistance to abscission, especially in “Nanyu.”

Trueman et al. (27) reported that natural fruit abscission is associated with declining fruit detachment force. Similarly, Deng et al. (8, 12) reported a strong relationship between grape fruit detachment force and berry abscission. In the current study, berry abscission increased gradually, concurrent with the reduction in detachment force, resulting in a negative correlation. Grape fruit detachment force is an index of berry adherence strength and is determined by the linking force between the berry and berry brush and the tensile strength of the abscission zone at the stalk–berry junction. Thus, the progress of berry abscission may be determined by a reduction in the detachment force of grape. However, the marked decrease in detachment force during storage may be explained by the observed cell dissolution, separation and breakdown in the abscission zones, as reported in ultrastructural changes in abscission zones of many fruits (13, 28).

## Conclusion

Our results showed that with prolonged storage time, both microstructural and ultrastructural alterations of the stalk–berry junction, berry brush, and pedicel occurred in “Muscat Kyoho,” “Kyoho,” and “Nanyu.” Under the stimuli of adverse environmental conditions, at the microstructural level, the cells of “Muscat Kyoho” and “Kyoho” became suberized and lignified before dissolving and gradually separating, forming intercellular cavities and producing an abscission zone. Furthermore, at the ultrastructural level, the cells of “Muscat Kyoho” and “Kyoho” became hydrolytic and liquated, and granular objects were observed in the cells at 6 days of storage, especially in “Muscat Kyoho.” With increasing storage duration, the size and density of microstructures and ultrastructures increased, with the alterations in “Nanyu” being the smallest.

The results of the present study establish that the abscission of grape berries proceeds through an abscission zone characterized by multiple cell microstructural or ultrastructural alterations in the stalk–berry junction, berry brush, and pedicel tissue cells. During the whole storage period, the detachment force of grape decreased, whereas berry abscission simultaneously increased. Of the three varieties, “Nanyu” appears to be best at delaying berry abscission, whereas “Kyoho” and “Muscat Kyoho” appear to be very susceptible to berry abscission.

Taken together, the results indicate that postharvest berry abscission induced microstructure and ultrastructure alterations in three types of table grapes, with the greatest alterations occurring in “Muscat Kyoho,” followed by “Kyoho” and then “Nanyu.” These alterations were directly related to berry abscission and correlated inversely with detachment force, since “Nanyu” maintained the highest detachment force and the lowest berry abscission during storage, followed by “Kyoho” and “Muscat Kyoho.”

## Data Availability Statement

All datasets generated for this study are included in the article/supplementary material.

## Author Contributions

All authors listed have made a substantial, direct and intellectual contribution to the work, and approved it for publication.

## Conflict of Interest

The authors declare that the research was conducted in the absence of any commercial or financial relationships that could be construed as a potential conflict of interest.
